# Associations between blood antioxidant levels and femoral neck strength

**DOI:** 10.1186/s12891-023-06370-5

**Published:** 2023-04-01

**Authors:** Peng Niu, Yongxi Liu, Yanfeng Zhang, Lei Li

**Affiliations:** 1 Department of spine and joint surgery, Nan Yang Second General Hospital, Nanyang City, Henan Province 473009 China; 2 The Quzhou Affiliated Hospital of Wenzhou Medical University, Quzhou People’s Hospital, Quzhou City, Zhejiang Province 324002 China

**Keywords:** Femoral neck fracture, Antioxidants, Femoral neck strength

## Abstract

**Background:**

Studies have confirmed that antioxidants contribute to a lower risk of osteoporosis, which is an independent factor for femoral neck fracture (FNF). However, the associations between blood antioxidant levels and femoral neck strength remain unclear.

**Objective:**

Our aim was to test the hypothesis that levels of blood antioxidants are positively associated with composite indices of bone strength in femoral neck, which integrate the bending strength index (BSI), compressive strength index (CSI), and impact strength index (ISI), in a population of middle-aged and elderly individuals.

**Methods:**

This cross-sectional study utilized data from the Midlife in the United States (MIDUS) study. Blood levels of antioxidants were measured and analyzed.

**Results:**

In total, data from 878 participants were analyzed. Results of Spearman correlation analyses indicated that blood levels of 6 antioxidants (total lutein, zeaxanthin, alpha-carotene, 13-cis-beta-carotene, trans-beta-carotene and total lycopene) were positively associated with CSI, BSI, or ISI in middle-aged and elderly individuals. Conversely, blood gamma-tocopherol and alpha-tocopherol levels were negatively associated with CSI, BSI, or ISI scores. Furthermore, linear regression analyses suggested that only blood zeaxanthin levels remained positively associated with CSI (odds ratio, OR 1.27; 95% CI: 0.03, 2.50; *p* = 0.045), BSI (OR, 0.54; 95% CI: 0.03–1.06; *p* = 0.037), and ISI (OR, 0.06; 95% CI: 0.00, 0.13; *p* = 0.045) scores in the study population after adjusting for age and sex.

**Conclusions:**

Our results indicated that elevated blood zeaxanthin levels were significantly and positively associated with femoral neck strength (CSI, BSI, or ISI) in a population of middle-aged and elderly individuals. These findings suggest that zeaxanthin supplementation may reduce FNF risk independently.

## Introduction

Osteonecrosis of the femoral head (ONFH) is one of the most common debilitating diseases, as it increases the risk of traumatic and nontraumatic fracture occurrence in the general population [1, 2]. Existing evidence in ONFH patients suggests that these individuals have lower bone density of the femoral head, which may be related to a reduction in the osteogenic differentiation of bone marrow stromal cells (BMSCs) and an inhibition of osteogenic gene expression [2, 3]. A recent study demonstrated that nontraumatic ONFH patients had lower bone mineral density (BMD) of the lumbar spine and femoral neck than that of healthy populations [1]. Although BMD is widely used to evaluate bone strength [4, 5], other studies indicate that BMD may only reflect 50%-70% of total bone strength [6, 7]. The comprehensive indices of femoral neck strength include the bending strength index (BSI), compressive strength index (CSI) and impact strength index (ISI) which are good indicators of femoral bone strength, a measure that is considered a predictor for femoral neck fracture (FNF) [8].

Oxidative stress promotes bone loss and remodeling via its impact on the regulation of osteoblast survival and differentiation and enhancement of inflammatory responses [9]. Conversely, antioxidants can inhibit oxidative stress and prevent the pathological process [10]. Existing evidence indicates that the consumption of antioxidant rich fruits and vegetables, such as apples, tomatoes, and oranges, is related to attenuations in bone mass loss, a factor impacting fracture risk, in postmenopausal women [11]. However, the beneficial effects of an increased antioxidant intake on bone strength are controversial. On the one hand, some epidemiological studies have reported that an increased intake of certain antioxidants, including vitamin C, vitamin E, and carotenoids, may confer benefits to BMD in premenopausal and postmenopausal females [12–14]. On the other hand, several studies have reported a positive correlation between higher intakes of vitamin C and higher risk of osteoporosis [15, 16]. Furthermore, previous evidence also indicated that antioxidants may produce bone-site specific beneficial effects on bone health [17]. for example, a previous study reported that a high β-carotene consumption was related to increased BMD of the femoral neck and total hip rather than other body parts in postmenopausal women [12].

This cross-sectional study was conducted using data from the Midlife in the United States (MIDUS) study. We aimed to investigate the associations between the 10 blood antioxidants and BSI, CSI and ISI in the middle-aged and elderly individuals.

## Methods

### Study participants

**Fig. 1:**
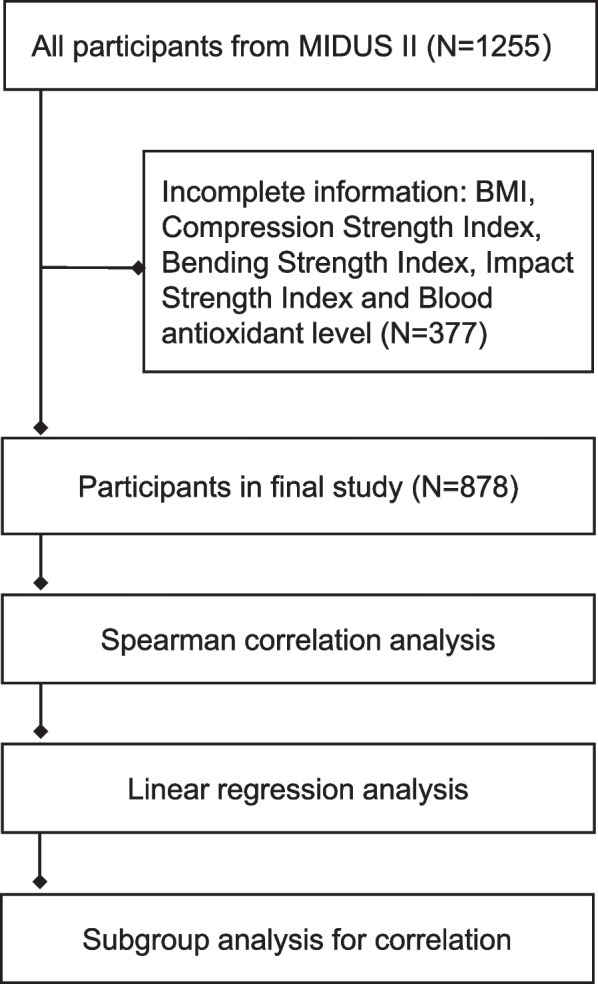
Flow chart displaying the final analysis of the included samples

The MIDUS Study mainly aimed to investigate the psychosocial and behavioral factors involved in age-related health conditions among a national sample of Americans [18, 19]. Data used in our study were obtained from participants in the Biomarker Project of MIDUS II (*N* = 1,255). The Biomarker Project recruited participants from the original MIDUS I cohort and aimed to measure various biological indicators in blood, urine, saliva, and other biological samples from 2004 to 2009 [19]. Additional details regarding the study methods and samples have been published elsewhere [18, 19]. Of the 1,255 participants in the Biomarker Project, we excluded data from 377 participants who had missing data on important variables. The final analysis from 878 participant samples were seen in Fig. 1. All methods were carried out in accordance with relevant guidelines and regulations, approval from appropriate Institutional Review Boards at the Midlife in the United States (MIDUS) study centers [three general clinical research centers (GCRC), including Georgetown University, the University of California at Los Angeles and the University of Wisconsin-Madison] was granted for this study and all participants gave informed consent before participation. We retrospectively analyzed MIDUS data from an open database (Inter-University Consortium for Political and Social Research).

### Blood measurements

Details for the collection methods of biological samples in the Biomarker Project have been previously described [20]. In summary, we measured and analyzed blood levels of 10 antioxidant markers (total lutein, zeaxanthin, beta-cryptoxanthin, 13-cis-beta-carotene, alpha-carotene, trans-beta-carotene, total lycopene, gamma-tocopherol, alpha-tocopherol, and retinol). Blood antioxidant measurements were obtained from fasting blood samples collected in the morning.

### Assessment of femoral neck strength

During each participants visit, the BMD values of the left hip and lumbar spine (L1-L4) were measured by DXA scans via Hologic 4500 (UCLA and Georgetown sites) technology or GE Healthcare Lunar Prodigy (Madison site). Femoral neck width (FNW) and femoral neck axis length (FNAL) were also measured from the hip scans using manufacturer guidelines. The composite indices of femoral neck strength (g/kg-m), including CSI, BSI, and ISI, were calculated by the following formulas [21]: 1) CSI = BMD × FNW/Weight; 2) BSI = (BMD × FNW^2^)/(FNAL × Weight); 3) ISI = (BMD × FNW × FNAL)/(Height × Weight).

### Statistical analyses

For research purposes, Spearman correlation analyses were preliminarily performed to examine the relationships between the 10 antioxidants and femoral neck strength (BSI, CSI and ISI). Then, linear regression analyses were conducted to assess the independent relationships among variables with antioxidants as independent variables and femoral neck strength indices as dependent variables (CSI, BSI and ISI) in the models. Age and sex have been reported to be related to bone mass loss [22], therefore the models were adjusted for age (Model 1) and sex (Model 2) successively. Standardized correlation coefficients and 95% confidence intervals (CIs) were reported for these models.

We used multiple linear regression analyses to explore the relationships between the 10 measured antioxidants and femoral neck strength, stratified by age, sex and BMI. We used the median age value to form a categorical variable for age (< 53 years and ≥ 53 years) and dichotomized BMI as < 24 kg/m^2^ or ≥ 24 kg/m^2^. Then, we tested for effect modification of age, sex and BMI on the associations. R version 4.0 and SPSS 25.0 were used for all analyses. The *P* value ≤ 0.05 represented a statistically significant difference.

## Results

### Characteristics of participants

A total of 878 samples were analyzed. As shown in Table 1, the age [53 (44–61) years], sex [male, 356 (40.55%)] and BMI [28.61 (25.07–32.97)] of our study sample were similar to the complete sample from the Biomarker Project. 377 Biomarker Project participants were mainly excluded from our analyses due to the presence of missing data for the femoral neck strength (BSI, CSI and ISI) and blood antioxidant variables. Hence, our study participants may have similar BDM [0.78 (0.66–1.02) gms/cm^2^], CSI [3.49 (3.05–4.03) g/kg-m], BSI [1.17 (1.00–1.36) g/kg-m] and ISI [0.20 (0.16–0.23) g/kg-m] values with the Biomarker Project sample. Similarly, the blood levels of antioxidants, including total lutein, zeaxanthin, beta-cryptoxanthin, 13-cis-beta-carotene, alpha-carotene, trans-beta-carotene, total lycopene, gamma-tocopherol, alpha-tocopherol, and retinol, were 0.20 (0.14–0.28) µmol/L, 0.05 (0.04–0.08) µmol/L, 0.17 (0.11–0.27) µmol/L, 0.05 (0.03–0.09) µmol/L, 0.06 (0.03–0.09) µmol/L, 0.37 (0.19–0.75) µmol/L, 0.39 (0.28–0.53) µmol/L, 3.66 (2.23–5.62) µmol/L, 25.95 (20.07–34.53) µmol/L and 1.58 (1.26–1.92) µmol/L, respectively.

**Table 1:** General characteristics of the study population

Variables	N (%) or Median (interquartile range)
Age (year)	53 (44–61)
Gender (male), n (%)	356 (40.55%)
BMI (kg/m^2)^	28.61 (25.07–32.97)
Femoral Neck bone mineral density (gms/cm^2^)	0.78 (0.66–1.02)
Compression Strength Index (g/kg-m)	3.49 (3.05–4.03)
Bending Strength Index (g/kg-m)	1.17 (1.00–1.36)
Impact Strength Index (g/kg-m)	0.20 (0.16–0.23)
**Blood markers**	
Blood total lutein (umol/L)	0.20 (0.14–0.28)
Blood zeaxanthin (umol/L)	0.05 (0.04–0.08)
Blood beta-cryptoxanthin (umol/L)	0.17 (0.11–0.27)
Blood 13-cis-beta-carotene (umol/L)	0.05 (0.03–0.09)
Blood alpha-carotene (umol/L)	0.06 (0.03–0.11)
Blood all trans-beta-carotene (umol/L)	0.37 (0.19–0.75)
Blood total lycopene (umol/L)	0.39 (0.28–0.53)
Blood gamma-tocopherol (umol/L)	3.66 (2.23–5.62)
Blood alpha-tocopherol (umol/L)	25.95 (20.07–34.53)
Blood retinol (umol/L)	1.58 (1.26–1.92)

*BMI:* Body mass index

### Spearman correlation analyses between blood levels of the measured antioxidants and bone strength of the femoral neck

We observed that elevated blood levels of total lutein, zeaxanthin, 13-cis-beta-carotene, alpha-carotene, trans-beta-carotene and total lycopene levels were positively associated with CSI (all P ≤ 0.05), and blood levels of gamma-tocopherol and alpha-tocopherol were negatively associated with CSI. Levels of circulating beta-cryptoxanthin and retinol were not associated with CSI (*P* > 0 0.05). Circulating concentrations of total lutein, zeaxanthin, beta-cryptoxanthin, 13-cis-beta-carotene, alpha-carotene, trans-beta-carotene and total lycopene were positively associated with BSI (all P ≤ 0.05). However, blood gamma-tocopherol and alpha-tocopherol concentrations were inversely related to BSI (all P ≤ 0.05). Similar results were present for ISI (Table 2).

**Table 2:** Spearman methods for correlation between blood antioxidant levels and bone strength of femoral neck

**Variables**	Femoral Neck bone mineral density (gms/cm^2^)	Compression Strength Index (g/kg-m)	Bending Strength Index (g/kg-m)	Impact Strength Index (g/kg-m)
Blood total lutein (umol/L)	-0.086*	0.122**	0.096**	0.104**
Blood zeaxanthin (umol/L)	0.002	0.110**	0.076*	0.073*
Blood beta-cryptoxanthin (umol/L)	-0.154**	0.055	0.094**	0.049
Blood 13-cis-beta-carotene (umol/L)	-0.095**	0.129**	0.093**	0.083*
Blood alpha-carotene (umol/L)	-0.145**	0.083*	0.083*	0.073*
Blood all trans-beta-carotene (umol/L)	-0.208**	0.075*	0.087*	0.086*
Blood total lycopene (umol/L)	0.001	0.068*	0.071*	0.073*
Blood gamma-tocopherol (umol/L)	0.022	-0.106**	-0.068*	-0.093**
Blood alpha-tocopherol (umol/L)	-0.109**	-0.108**	-0.066*	-0.068*
Blood retinol (umol/L)	-0.068*	-0.041	-0.033	0.011

Spearman correlation analysis, * < 0.05; ** < 0.01

### Adjusted associations between blood antioxidant levels and bone strength of the femoral neck

The results of our age and sex adjusted linear regression analyses, as shown in Table 3, confirmed that elevated blood zeaxanthin (*r* = 1.27; 95% CI: 0.03, 2.50; *P* = 0.045) and 13-cis-beta-carotene levels (*r* = 0.11; 95% CI: 0.28, 1.94; *P* = 0.009) were associated with increased CSI in the femoral neck, whereas elevated blood gamma-tocopherol (*r* = -0.03; 95% CI: -0.05, -0.01; *P* = 0.006) and alpha-tocopherol (*r* = -0.01; 95% CI: -0.01, -0.00; *P* = 0.024) levels were associated with lower CSI. Moreover, we found that blood total lutein (*r* = 0.19; 95% CI: 0.03, 0.35; *P* = 0.024), zeaxanthin (*r* = 0.54; 95% CI: 0.03, 1.06; *P* = 0.037), beta-cryptoxanthin (*r* = 0.22; 95% CI: 0.09, 0.36; *P* = 0.001), 13-cis-beta-carotene (*r* = 0.37; 95% CI: 0.02, 0.71; *P* = 0.036) and alpha-carotene (*r* = 0.28; 95% CI: 0.02, 0.54; *P* = 0.035) levels were positively associated with BSI. Finally, the results of the linear regression analyses were similar for ISI, as shown in Table 3.

**Table 3:** Linear regression analysis for correlation between blood antioxidant levels and bone strength of femoral neck

Variables	Model 1		Model 2	
	Sβ (95% CI)	*P* Value	Sβ (95% CI)	*P* Value
**Femoral Neck bone mineral density (gms/cm**^**2**^ **)**				
Blood total lutein (umol/L)	-0.21 (-0.34, -0.09)	< 0.001**	-0.17 (-0.30, -0.05)	0.007**
Blood zeaxanthin (umol/L)	-0.20 (-0.60, 0.20)	0.322	-0.16 (-0.55, 0.23)	0.423
Blood beta-cryptoxanthin (umol/L)	-0.14 (-0.25, -0.04)	0.007**	-0.12 (-0.22, -0.02)	0.022*
Blood 13-cis-beta-carotene (umol/L)	-0.28 (-0.55, -0.01)	0.039*	-0.21 (-0.47, 0.05)	0.119
Blood alpha-carotene (umol/L)	-0.24 (-0.44, -0.04)	0.020*	-0.21 (-0.41, -0.02)	0.035*
Blood all trans-beta-carotene (umol/L)	-0.03 (-0.06, -0.01)	0.007**	-0.03 (-0.05, -0.00)	0.048*
Blood total lycopene (umol/L)	-0.01 (-0.10, 0.08)	0.814	-0.04 (-0.13, 0.05)	0.338
Blood gamma-tocopherol (umol/L)	0.00 (-0.00, 0.01)	0.268	0.00 (-0.00, 0.01)	0.509
Blood alpha-tocopherol (umol/L)	-0.00 (-0.00, 0.00)	0.153	-0.00 (-0.00, 0.00)	0.834
Blood retinol (umol/L)	-0.03 (-0.06, -0.00)	0.024*	-0.02 (-0.05, 0.01)	0.123
**Compression Strength Index (g/kg-m)**				
Blood total lutein (umol/L)	0.32 (-0.06, 0.71)	0.102	0.38 (-0.01, 0.77)	0.059
Blood zeaxanthin (umol/L)	1.28 (0.05, 2.52)	0.042*	1.27 (0.03, 2.50)	0.045*
Blood beta-cryptoxanthin (umol/L)	0.20 (-0.12, 0.53)	0.220	0.23 (-0.09, 0.56)	0.165
Blood 13-cis-beta-carotene (umol/L)	1.15 (0.32, 1.98)	0.007**	1.11 (0.28, 1.94)	0.009**
Blood alpha-carotene (umol/L)	0.64 (0.01, 1.27)	0.046*	0.59 (-0.04, 1.21)	0.067
Blood all trans-beta-carotene (umol/L)	0.05 (-0.03, 0.13)	0.209	0.05 (-0.03, 0.13)	0.216
Blood total lycopene (umol/L)	0.07 (-0.21, 0.35)	0.627	0.06 (-0.22, 0.34)	0.661
Blood gamma-tocopherol (umol/L)	-0.02 (-0.04, -0.01)	0.014*	-0.03 (-0.05, -0.01)	0.006**
Blood alpha-tocopherol (umol/L)	-0.01 (-0.01, -0.00)	0.009**	-0.01 (-0.01, -0.00)	0.024*
Blood retinol (umol/L)	-0.07 (-0.16, 0.01)	0.076	-0.05 (-0.14, 0.03)	0.192
**Bending Strength Index (g/kg-m)**				
Blood total lutein (umol/L)	0.18 (0.02, 0.34)	0.029*	0.19 (0.03, 0.35)	0.024*
Blood zeaxanthin (umol/L)	0.59 (0.09, 1.10)	0.021*	0.54 (0.03, 1.06)	0.037*
Blood beta-cryptoxanthin (umol/L)	0.22 (0.09, 0.35)	0.001**	0.22 (0.09, 0.36)	0.001**
Blood 13-cis-beta-carotene (umol/L)	0.46 (0.12, 0.80)	0.008**	0.37 (0.02, 0.71)	0.036*
Blood alpha-carotene (umol/L)	0.35 (0.10, 0.61)	0.007**	0.28 (0.02, 0.54)	0.035*
Blood all trans-beta-carotene (umol/L)	0.04 (0.01, 0.07)	0.019*	0.03 (-0.00, 0.06)	0.080*
Blood total lycopene (umol/L)	0.05 (-0.06, 0.17)	0.350	0.08 (-0.04, 0.19)	0.200
Blood gamma-tocopherol (umol/L)	-0.01 (-0.01, 0.00)	0.117	-0.01 (-0.02, 0.00)	0.064
Blood alpha-tocopherol (umol/L)	-0.00 (-0.00, 0.00)	0.200	-0.00 (-0.00, 0.00)	0.286
Blood retinol (umol/L)	-0.04 (-0.07, -0.01)	0.018*	-0.03 (-0.06, 0.00)	0.094
**Impact Strength Index (g/kg-m)**				
Blood total lutein (umol/L)	0.02 (-0.00, 0.04)	0.060	0.02 (0.00, 0.04)	0.050
Blood zeaxanthin (umol/L)	0.07 (0.01, 0.13)	0.025*	0.06 (0.00, 0.13)	0.045
Blood beta-cryptoxanthin (umol/L)	0.02 (0.00, 0.03)	0.049*	0.02 (0.00, 0.03)	0.043*
Blood 13-cis-beta-carotene (umol/L)	0.05 (0.01, 0.09)	0.024*	0.04 (-0.01, 0.08)	0.097
Blood alpha-carotene (umol/L)	0.05 (0.02, 0.08)	0.003**	0.04 (0.01, 0.07)	0.020*
Blood all trans-beta-carotene (umol/L)	0.00 (0.00, 0.01)	0.023	0.00 (-0.00, 0.01)	0.101
Blood total lycopene (umol/L)	0.01 (-0.00, 0.03)	0.083	0.02 (0.00, 0.03)	0.036
Blood gamma-tocopherol (umol/L)	-0.00 (-0.00, -0.00)	0.002	-0.00 (-0.00, -0.00)	< 0.001
Blood alpha-tocopherol (umol/L)	-0.00 (-0.00, -0.00)	0.021	-0.00 (-0.00, -0.00)	0.035
Blood retinol (umol/L)	-0.00 (-0.01, 0.00)	0.281	-0.00 (-0.00, 0.00)	0.714

Model 1: Adjusted for age

Model 2: Adjusted for age and gender

^*^ < 0.05; ** < 0.01

### Subgrouping analyses on the associations between blood zeaxanthin levels and bone strength of the femoral neck

**Fig. 2:**
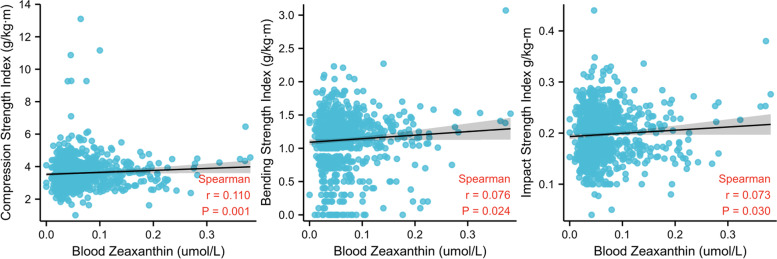
Scatter diagram depicting the associations between blood zeaxanthin and the femoral neck strength (BSI, CSI and ISI)

The above results showed that only elevated blood zeaxanthin levels were positively and significantly correlated with CSI, BSI, and ISI, respectively. Thus, we further analyzed scatter plots and found that higher blood zeaxanthin levels may have contributed to higher composite indices of femoral neck strength (Fig. 2). Furthermore, we conducted subgrouping analyses to evaluate the effects of age, sex, and BMI on the relationship between blood zeaxanthin levels and bone strength of the femoral neck. Interestingly, although the interaction effects for age, sex, and BMI were not statistically significant (all interaction *P* values ≥ 0.05), as shown in Table 4, we still found that elevated blood zeaxanthin levels were associated with higher CSI, BSI and ISI in female participants aged ≥ 53 years or with a BMI ≥ 24 kg/m^2^.

**Table 4:** Grouping analysis for correlation between blood zeaxanthin levels and bone strength of femoral neck

Variables		Femoral Neck bone mineral density (gms/cm^2^)			Compression Strength Index (g/kg-m)			Bending Strength Index (g/kg-m)			Impact Strength Index (g/kg-m)		
		Sβ (95% CI)	*P* Value	P*	Sβ (95% CI)	*P* Value	P*	Sβ (95% CI)	*P* Value	P*	Sβ (95% CI)	*P* Value	P*
Age (year)	< 53	-0.20 (-0.86, 0.46)	0.561	0.720	0.46 (-1.52, 2.43)	0.651	0.173	0.34 (-0.41, 1.10)	0.375	0.349	0.02 (-0.07, 0.12)	0.629	0.171
	≥ 53	-0.05 (-0.53, 0.43)	0.842		2.19 (0.62, 3.76)	0.006		0.83 (0.15, 1.52)	0.018		0.11 (0.03, 0.19)	0.006	
Gender	Male	-0.04 (-0.84, 0.76)	0.926	0.767	1.01 (-0.82, 2.84)	0.281	0.676	0.67 (-0.18, 1.52)	0.124	0.871	0.03 (-0.08, 0.15)	0.566	0.397
	Female	-0.17 (-0.59, 0.26)	0.450		1.58 (0.07, 3.09)	0.041		0.58 (-0.06, 1.22)	0.076		0.09 (0.02, 0.16)	0.014	
BMI (kg/m2)	< 24	-0.08 (-0.82, 0.66)	0.835	0.879	0.55 (-1.49, 2.59)	0.598	0.586	0.14 (-0.75, 1.03)	0.758	0.408	0.04 (-0.08, 0.17)	0.495	0.741
	≥ 24	-0.15 (-0.60, 0.29)	0.499		1.39 (0.06, 2.72)	0.041		0.66 (0.09, 1.23)	0.022		0.07 (0.00, 0.13)	0.035	

Adjusted for age and gender

P* Interaction *P* value

## Discussion

There are many studies investigating the associations between antioxidants and bone health. For instance, it has been reported that greater serum carotenoid and lutein concentrations are associated with higher BMD in Chinese adults [23], and an adequate intake of vegetables may reduce the risk of osteoporotic fractures among elderly men. The antioxidation of carotenoids may counteract the mechanism of osteoporosis related to leanness [24]. Consistent with carotenoids, elevations in serum levels of lutein and zeaxanthin play a role in bone health [25]. Oxidative stress is associated with lower BMD, which is more pronounced in individuals with low serum levels of vitamin E, as are often observed in older men [26]. Consistently, our results also showed that increased antioxidant levels were cross-sectionally associated with elevated indices of femoral neck strength (CSI, BSI, and ISI) in a representative sample of Americans. A positive correlation between zeaxanthin levels and femoral neck strength (CSI, BSI, and ISI) was also observed after adjustment for age and sex. One recent meta-analysis concluded that the role of vitamin A or its derivatives on BMD remain unclear, although most of the included studies showed a favorable effect of vitamin A on BMD [27]. This is also consistent with our results which showed no significant associations between blood retinol levels and BMD or bone strength of the femoral neck.

Antioxidants are important substances for eliminating free radicals. It can reduce the oxidative stress responses in the body and increase BMD. Therefore, consuming adequate amounts of fruits and vegetables can reduce the risk of osteoporosis and its complications, such as pain and fracture [28]. Recent epidemiological evidence concluded that relatively high intakes of antioxidants, including carotenoids [6], vitamin E, vitamin C, and flavonoids were linked to an increased BMD in postmenopausal women [12–14, 29]. A recent study reported a causal link between increased circulating α-tocopherol and elevated BMD [30]. Another observational study also found that total dietary antioxidant capacity of as inversely associated with the risk of osteoporosis in postmenopausal women and positively associated with bone mass in both pre- and postmenopausal women [31]. These previous observational studies consistently asserted that the intake of antioxidants may strongly impact BMD in femoral neck. Conversely, our study showed negative associations between blood total lutein, beta-cryptoxanthin, alpha-carotene and all trans-beta-carotene levels and BMD, after adjusting for age and sex. There were no significant associations between blood gamma-tocopherol or alpha-tocopherol concentrations and BMD. A possible reason for this finding is that we included a sample of generally healthy participants in our analyses rather than a specific population, such as postmenopausal women or elderly individuals. Another important reason may be that we measured the participants’ circulating antioxidant levels, rather than assessing fruit and vegetable intake, which was a commonly used method in previous studies. Our results showed that the circulating levels of antioxidants were negatively correlated or uncorrelated with the BMD of the femoral neck, which is an interesting phenomenon and needs to be confirmed in future investigations.

Osteoporosis typically occurs in individuals who are 50 years of age or older. A decrease in bodily hormone levels, particularly in postmenopausal women, leads to the proliferation of osteoclasts and the acceleration in bone loss. Increased fruit and vegetable intake has been related to bone mineral content in premenopausal women [28]. Further, the increased intake of vitamin C was related to higher femur BMD in premenopausal women [32]. These studies suggest that antioxidant intake confers clear benefits on BMD in premenopausal women. Consistently, our study also suggested that blood antioxidant levels were positively associated with femoral neck strength (CSI, BSI and ISI) in female subjects aged ≥ 53 years. These findings confirm that the consumption of antioxidant rich food is beneficial to increase femoral neck strength and may subsequently prevent FNF. Interestingly, we also observed a positive correlation between blood antioxidant levels and femoral neck strength in participants with overweight or obesity (BMI ≥ 24 kg/m^2^), but not in participants with a normal BMI (BMI < 24 kg/m^2^). This may suggest that supplementation with antioxidants is beneficial to increase the strength of the femoral neck in individuals with an overweight or obese BMI.

Compared with previous observational studies, our study has several advantages. Our data were obtained from the MIDUS study, which enrolled a representative sample of Americans. The MIDUS II Biomarker Project conducted high-quality assessments on blood samples, which were obtained from a large sample of the general population. Our study included measures of almost all lipid soluble antioxidants, which allowed us to comprehensively analyze the relationships between blood antioxidants and bone strength. In addition to BMD, we also analyzed femoral neck strength indices (CSI, BSI, and ISI), making this the first study to analyze the relationship between blood antioxidants and femoral neck fracture-related indicators. Importantly, the retrospective analyses conducted in the present study did impose some limitations. First, the inherent disadvantages of cross-sectional studies made it difficult to assess causal associations between antioxidants and both BMD and femoral neck bone strength, although the pathological mechanism by which reducing oxidative stress may attenuate bone mass loss is well characterized. Second, the statistical models were adjusted for age, sex, and BMI, but there are other known and unknown confounding variables that were not measured or included in the analyses. For example, we did not include information on disease history, including such conditions as hypertension, coronary heart disease and diabetes, in the analyses. Additionally, data related to menopausal status in females and drug treatment status (e.g., vitamin D supplementation or other drug treatments that may cause osteoporosis) were not included in the analyses. Therefore, the results may be impacted by these factors. Third, 377 samples from the MIDUS II Biomarker Project (*N* = 1,225) were not analyzed due to missing data, and only data from 878 subjects were included in our study, potentially producing an offset of the sample section.

## Conclusion

We comprehensively conducted a cross-sectional analysis of the relationship between blood levels of 10 antioxidants and bone strength of the femoral neck. Our results indicate that increasing blood levels of antioxidants, especially zeaxanthin, may increase femoral neck strength (CSI, BSI, and ISI). These findings supported that antioxidant supplementation can further reduce FNF risk.

## Data Availability

The data used in the present study are publicly available through the Inter-University Consortium for Political and Social Research (ICPSR): www.icpsr.umich.edu/web/ICPSR/studies/29282.
