# Correction: Associations between blood antioxidant levels and femoral neck strength

**DOI:** 10.1186/s12891-023-06535-2

**Published:** 2023-05-26

**Authors:** Peng Niu, Yongxi Liu, Yanfeng Zhang, Lei Li

**Affiliations:** 1 Department of spine and joint surgery, Nan Yang Second General Hospital, Nanyang City, 473009 Henan Province China; 2 The Quzhou Affiliated Hospital of Wenzhou Medical University, Quzhou People’s Hospital, Quzhou City, 324002 Zhejiang Province China

**Correction:** ***BMC Musculoskelet Disord*** **24, 252 (2023)**

10.1186/s12891-023-06370-5

Following publication of the original article [1], the authors would like to insert an article note (The affiliation “*The Quzhou Affiliated Hospital of Wenzhou Medical University, Quzhou People’s Hospital, Quzhou City, 324002, Zhejiang Province, China”* of Dr. Lei Li is the main or first institution of this research.) to emphasize the main institution of this research.

The original article [1] has been corrected.
